# Does the Effort of Processing Potential Incentives Influence the Adaption of Context Updating in Older Adults?

**DOI:** 10.3389/fpsyg.2017.01969

**Published:** 2017-11-09

**Authors:** Hannah Schmitt, Jutta Kray, Nicola K. Ferdinand

**Affiliations:** Department of Psychology, Saarland University, Saarbrücken, Germany

**Keywords:** motivational incentives, proactive/reactive cognitive control, context processing, aging, ERPs

## Abstract

A number of aging studies suggest that older adults process positive and negative information differently. For instance, the socioemotional selectivity theory postulates that older adults preferably process positive information in service of emotional well-being (Reed and Carstensen, 2012). Moreover, recent research has started to investigate whether incentives like gains or losses can influence cognitive control in an ongoing task. In an earlier study (Schmitt et al., 2015), we examined whether incentive cues, indicating potential monetary gains, losses, or neutral outcomes for good performance in the following trial, would influence older adults’ ability to exert cognitive control. Cognitive control was measured in an AX-Continuous-Performance-Task (AX-CPT) in which participants had to select their responses to probe stimuli depending on a preceding context cue. In this study, we did not find support for a positivity effect in older adults, but both gains and losses led to enhanced context processing. As the trial-wise presentation mode may be too demanding on cognitive resources for such a bias to occur, the main goal of the present study was to examine whether motivational mindsets, induced by block-wise presentation of incentives, would result in a positivity effect. For this reason, we examined 17 older participants (65–76 years) in the AX-CPT using a block-wise presentation of incentive cues and compared them to 18 older adults (69–78 years) with the trial-wise presentation mode from our earlier study (Schmitt et al., 2015). Event-related potentials were recorded to the onset of the motivational cue and during the AX-CPT. Our results show that (a) older adults initially process cues signaling potential losses more strongly, but later during the AX-CPT invest more cognitive resources in preparatory processes like context updating in conditions with potential gains, and (b) block-wise and trial-wise presentation of incentive cues differentially influenced cognitive control. When incentives were presented block-wise, the above described valence effects were consistently found. In contrast, when incentives were presented trial-wise, the effects were mixed and salience as well as valence effects can be obtained. Hence, how positive and negative incentive cues influence cognitive control in older adults is dependent on demands of cue processing.

## Introduction

In daily life, motivation and cognition interact in many ways to determine goal-directed behavior. Motivational influences on goal-directed behavior become especially important in old age, when failures of cognitive functioning dramatically affect individual autonomy (Schmitt and Kray, 2015). The aim of the present study was to investigate how a specific motivational mindset, i.e., an induced cognitive orientation toward positive (anticipating monetary gains) and negative events (anticipating monetary losses), modulates cognitive control functioning in older adults. We were specifically interested in whether a positive or negative mindset would differentially influence cognitive control functioning.

In their dual-mechanisms-of-control (DMC) theory, Braver and Barch (2002) suggested that age-related impairments in a variety of cognitive tasks can be ascribed to age-related neuro-biological changes in the prefrontal cortex (PFC) and the dopamine (DA) system (Suhara et al., 1991; Raz, 2000; Bäckman and Farde, 2005) associated with the ability to process and update context information. Although the definition of “context” might differ between research domains (Ibañez and Manes, 2012), context information in the DMC theory refers to task instructions, rules and goals that, akin to a mindset, are actively maintained in memory to optimally adjust behavior (cf. Braver et al., 2005). In particular, the DMC theory proposes an age-differential pattern of cognitive control associated with processing and updating context information. It assumes that younger adults predominantly engage in proactive control, i.e., an early selection, updating, and maintenance of contextual information to bias subsequent cognitive processing. Functional magnetic resonance imaging (fMRI) studies indeed show that proactive control in younger adults is related to sustained and anticipatory activity of the lateral PFC and that the midbrain dopamine system supports the updating of PFC-representations in advance preparation prior to task execution (Braver and Barch, 2002; Braver and Bongiolatti, 2002; Bugg, 2013). Older adults, in turn, more heavily rely on reactive control, i.e., a bottom-up manner of processing contextual information when needed to resolve interference in an ongoing task. Accordingly, reactive control in older adults was associated with a transient activation of the lateral PFC as well as an activation of the anterior cingulate cortex (ACC) serving the detection of conflict during task execution (Paxton et al., 2006; Braver et al., 2008; Jimura and Braver, 2009; Braver, 2012).

Using the high temporal resolution of event-related potential (ERPs), our previous studies support differential time courses of pro- and reactive control during context processing across age groups (Schmitt et al., 2014a,b). In these studies, we applied a variant of the AX-CPT (cf. Lenartowicz et al., 2010), consisting of context-dependent (c-dep) and context-independent (c-indep) trials. On c-dep trials, correct responses to one of two probes are directly dependent on a preceding cue. On c-indep trials, the correct response to one of two probes is always the same, irrespective of the preceding context cue. Accordingly, context processing should occur to a lesser extent on c-indep trials as the cue is irrelevant for inferring the correct response. Thus, less cognitive control is needed. Our results in the cue interval indicated that older adults updated more task-relevant context cue information as reflected in a larger P3b (Donchin and Coles, 1988) whenever the identity of the context cue changed, while younger adults showed more updating only in the demanding c-dep trials. We also found a context effect in the contingent negative variation (CNV) that was of the same size for older and younger adults, indicating that more task set maintenance is necessary (Kray et al., 2005) on c-dep than on c-indep trials. Thus, there were age-related differences in how effectively proactive control is applied during context cue processing (Schmitt et al., 2014a). Moreover, older adults exhibited a larger amplitude of the N450 to c-dep than c-indep trials in the probe interval. The N450 component has been interpreted as reflecting conflict processing in the ACC and behavioral adaptation during task execution (West and Alain, 2000a; West, 2004). Thus, larger amplitudes of the N450 to c-dep than c-indep trials may indicate the greater need to resolve response conflict at the time the probe is presented as predicted by a reactive control style (Schmitt et al., 2014b).

Recently, research has investigated the flexible engagement of pro- and reactive control in adapting to various environmental conditions, such as motivational goals. Motivational stimuli signaling potential reward (see Gruber and Otten, 2010; Halsband et al., 2012), have been shown to foster the gating of upcoming task-relevant context information to the PFC (D’Ardenne et al., 2012), and to improve proactive context updating in younger adults (Locke and Braver, 2008; Chiew and Braver, 2013). In contrast, the impact of motivationally negative stimuli (e.g., prospective punishment) on cognitive control processing has been widely neglected (Engelmann and Pessoa, 2007). Similarly, not much is known about how cognitive control functions can be influenced by motivational incentives in old age. In one of our earlier studies, we examined whether different motivational cues modulate the time course of context processing in younger and older adults. In this study, participants performed an adapted version of the AX-CPT that included a trial-wise presentation of motivational cues either announcing a potential monetary gain, loss, or neutral outcome depending on individual performance (Schmitt et al., 2015). The ERP data locked to the motivational cues showed larger P2 and P3b amplitudes to salient gain and loss cues as compared to neutral cues for older adults, indicating more automatic capture of attention (Carretié et al., 2004; for a review, see Olofsson et al., 2008) and more updating of task-relevant information (Donchin and Coles, 1988; Briggs and Martin, 2009; Krebs et al., 2013) in the case of salient motivational cue information. Similarly, in the context cue epoch, older adults displayed larger P3b amplitudes for c-dep than c-indep trials for motivationally salient gain *and* loss cues. Gains and losses also resulted in a temporally prolonged probe-locked P3b, suggesting that older adults invested more in context updating and task reconfiguration during response preparation and execution. Hence, the results suggested that motivationally salient information led to an early cue-locked representation of context conditions in older adults, indicating that potential gains and losses elicit a shift toward proactive control and also strengthen reactive control processes.

However, the finding that older adults did not differentiate between conditions with potential gains and losses was surprising as there is evidence from other cognitive domains showing a preference toward remembering positive information (Mather, 2006). According to the age-related positivity effect, older adults direct more attention to and have better memory of positive relative to negative and neutral information compared to younger adults (Mather and Carstensen, 2005). This effect has been explained in the framework of the socioemotional selectivity theory (SST), postulating that emotional satisfaction and well-being, for instance by remembering more positive events, is prioritized when future time horizons are restricted (Reed and Carstensen, 2012). In line with this idea, Samanez-Larkin et al. (2007) demonstrated that older adults show age-related impairments (reduced striatal and insular activation) during the processing of potential losses, but not during processing of potential gains. Similarly, ERP studies found that older adults’ are less affected by negative feedback and rely more on positive feedback during learning (e.g., Eppinger et al., 2008; Pietschmann et al., 2011). However, results are far from consistent. For instance, Eppinger et al. (2013) found age-related impairments in learning from monetary rewards but not losses and Frank and Kong (2008) found that old–old adults (mean age = 77 years) as opposed to young–old adults (mean age = 67 years) were better in learning to avoid stimuli that had been coupled with negative feedback before (see also Hämmerer et al., 2010). Consequently, it is not quite clear to what extent the positivity-effect generalizes to other domains of cognitive functioning. Also, there is evidence for the absence of a positivity effect when less resources for cognitive control are available for the task at hand (Mather and Knight, 2005; Knight et al., 2007; Gorlick et al., 2013) or in individuals with low levels of cognitive control functioning (Reed and Carstensen, 2012). Thus, in our previous study (Schmitt et al., 2015), in which the motivational cue information switched on a trial-to-trial basis, older adults might have been strongly engaged in adapting behavior to relevant motivational cue information on each trial, leaving less cognitive resources for processing the valence of motivational cues and to adjust behavior accordingly.

The main goal of the present study was to investigate whether the valence of motivational information (i.e., cue stimuli indicating potential gains or losses) differently influences cognitive control functioning in older adults when cognitive demands on cue processing are reduced. To this end, older adults performed the AX-CPT containing motivational cues, indicating potential positive (i.e., reward), negative (i.e., punishment) or neutral outcomes, but these motivational cues did not change within the actual task block. We induced a motivational mindset by presenting motivational cues in a block-wise fashion and compared this to the trial-wise cue presentation mode from our earlier study (Schmitt et al., 2015). Motivational effects, i.e., salience and valence effects were operationalized by comparing performance and ERP components on neutral against motivational gain *and* loss blocks, and between loss and gain blocks, respectively (see Bromberg-Martin et al., 2010). Cognitive control functioning was operationalized by examining c-dep and c-indep trials in the AX-CPT (Braver, 2012). Our reasoning was that this block-wise presentation of incentive cues should reduce updating requirements and may lead to the emergence of a valence effect as older adults have sufficient cognitive resources to direct attention toward the processing of positive cue information (Reed and Carstensen, 2012). We assumed that if the preference to process positive information in older adults indeed depends on the amount of cognitive resources available, then older adults may show enhanced attention (as indexed by a larger P2) and more updating (as reflected in a larger P3b) after positive relative to negative and neutral cue information in the motivational cue epoch (i.e., a valence effect), when cognitive demands are reduced by a block-wise cue presentation. We further expected this positivity effect to transfer to the AX-CPT, indicating a selective strengthening of context updating when positive information is anticipated. Here, the valence effect should be reflected in an enhanced difference between c-dep and c-indep trials in P3b and CNV amplitudes in the context cue epoch and in N450 amplitudes in the probe epoch on monetary gain trials only.

## Materials and Methods

### Participants

Twenty-three older adults, recruited from a subject pool at Saarland University, took part in the study. Three participants had to be excluded because their latencies and/or error rates (ERs) in the AX-CPT were more than three standard deviations above the group means, indicating that they either did not fully understand the task or the task was too difficult for them. Two further participants were excluded because the number of artifact-free trials for EEG analysis was too low (less than 16) and one because he had already participated in an earlier version of this experiment and had been invited by accident. Therefore, the final sample included 17 older adults (mean age = 71.8 years, age range = 65–76 years, 53% females). Informed consent was obtained from each participant. The study was approved by the local ethics committee at Saarland University and conducted in accordance with the Declaration of Helsinki. Subjects were paid eight Euros per hour with an additional reimbursement based on the money won in the motivational blocks (see **Table 1**). All participants had normal or corrected-to-normal vision, no signs of color-blindness, and were free of self-reported neurological or psychological disorders. All subjects also performed three psychometric tests of cognitive functioning: The Digit-Symbol-Substitution Test (DSST, adapted from Wechsler, 2008) measured speed of processing, the Counting Span task (CS; adapted from Unsworth et al., 2005) served as a WM span measure, and the Spot-a-word test (Lehrl, 1977; Lindenberger et al., 1993) was used as an indicator of vocabulary. The results of the three cognitive control variables and the characteristics of the sample are displayed in **Table 1**. All participants performed well within their normal age range (cf. Myerson et al., 2003; Kray et al., 2008; Elliot et al., 2011; Ferdinand and Kray, 2013).

**Table 1:** Sample characteristics and results of psychometric measures (means and standard deviations).

	Older adults	
Measure	*M*	*SD*
*n*	17	
Mean age (years)	71.8	3.0
Age range (years)	65–76	
Gender distribution (% female)	43%	
Digit symbol substitution test	51.1	6.5
Counting span	29.7	7.6
Spot-a-word	28.7	3.4
Money won (euro)	7.2	1.3

The participants described above performed the AX-CPT with incentive cues presented in a block-wise fashion (block-wise group) and were compared with the older participants from our previous experiment (for a detailed sample description, see Schmitt et al., 2015), who performed the AX-CPT with incentive cues presented in a trial-wise fashion (trial-wise group).

### Tasks and Stimuli

**FIGURE 1:**
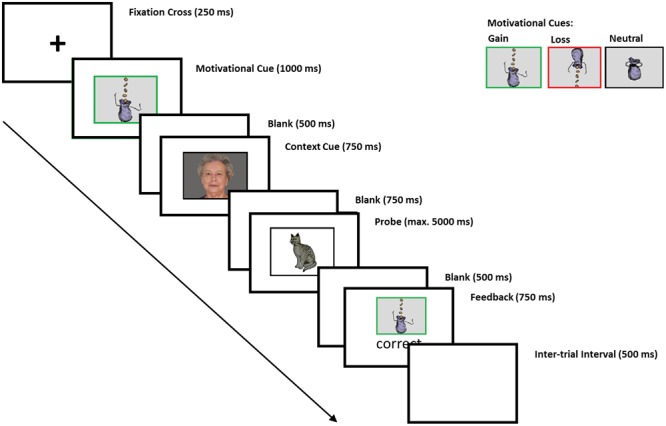
Assignment of cues and probes to correct response keys on c-dep and c-indep trials. On c-dep trials, the correct responses to probes (as indexed by the pictures of the bird and the cat) after presentation of the cues (as indexed by the pictures of the younger woman and the older man) are exactly reversed for the two cues; i.e., participants have to press the left response key in case the bird follows the younger woman, whereas they have to press the right key in case the bird follows the older man. On c-indep trials, correct responses to the probes (as indexed by the pictures of the fish and the rabbit) are identical for both cues (as indexed by the pictures of the younger man and the older woman); i.e., participants always have to press the left key if they see the picture of the fish and the right key if they see the picture of the rabbit (Facial pictures by Minear and Park, 2004, pictures of animals by Rossion and Pourtois, 2004).

A modified version of the AX-CPT was applied using E-Prime 2 (Psychology Software Tools) in which subjects saw cue-probe combinations (for a detailed description, see Schmitt et al., 2015; adapted from Lenartowicz et al., 2010; see **Figure 1**). We used four pictures of young and old men and women (Minear and Park, 2004) as context cue stimuli and four pictures of animals (i.e., rabbit, bird, cat, and fish from the database by Rossion and Pourtois (2004)) as probe stimuli. On c-dep trials, correct responses to subsequent probes were dependent on the preceding context cue. For instance, subjects were instructed to press the *left* key when the picture of the *bird* followed the picture of the *young woman* and the *right* key when the picture of the *cat* followed the picture of the *young woman*. These stimulus-response assignments were reversed when the “bird” and the “cat” followed the picture of the old man. On c-indep trials, correct responses to probes were independent of the preceding context cue. Subjects were instructed to press the *left* key when the picture of the *rabbit* followed the picture of the *old woman* and the *right* key when the picture of the *fish* followed the picture of the *old woman.* The same stimulus-response assignments were required when the “fish” and the “rabbit” followed the picture of the young man. Note that subjects were instructed to respond to four cue-probe combinations and were not informed about the two different trial types (i.e., c-dep and c-indep trials).

Prior to the cue-probe combinations, participants received three variants of motivational cues (see **Figure 1**). The “neutral” motivational cue was a picture of a (closed) moneybag surrounded by a black frame. The motivational gain cue was a picture of a gain moneybag, i.e., money falling into the bag, which was surrounded by a green frame. The loss cue was a picture of a loss moneybag, showing money dropping out of the bag, was surrounded by a red frame. Participants were instructed that the gain cue indicated the possibility to win money if they responded correctly, and that the loss cue indicated the risk to lose money if they responded incorrectly. On neutral blocks, the monetary value remained constant irrespective of the response accuracy. Stimuli were presented in a 3.5 cm × 5.5 cm black frame on the center of a 24 in. monitor on a gray background and participants’ viewing distance was approximately 80 cm. The assignment of context-cue conditions to response keys was pseudo-random across participants with the only constraint that a young and an old facial picture were presented in both trial types for each participant. This led to four context conditions that were equally assigned to male and female subjects.

### Procedure

Subjects first filled in an informed consent, a demographic and health questionnaire, and a handedness rating (Oldfield, 1971). Afterward, they were tested on the three control variables described above and the AX-CPT. All subjects were initially taught the meaning of the motivational cues and then performed three practice blocks of the AX-CPT. To familiarize participants with both trial types, the first practice block consisted of c-indep trials only, the second block of c-dep trials only, and the third practice block included both c-dep and c-indep trials. In case subjects did not understand the task during the first practice run, practice blocks were repeated. None of the subjects had more than two repetitions of any practice block.

Each participant performed the AX-CPT under three motivational mind-sets that were induced by block-wise instructions for three cue conditions (neutral, gain, or loss). In the first three task blocks, the three motivational conditions (neutral, gain, and loss) were presented successively in a random order. This order was then exactly repeated in the next three blocks, yielding a total of six motivational blocks. Each motivational block consisted of 48 c-dep and 48 c-indep trials. As a result, the AX-CPT consisted of a total of 96 c-dep and c-indep trials for each of the three motivational conditions (neutral, gain, and loss). After each block, a rest period was mandatory. In the rest period, participants received feedback about the amount of money they had earned so far, which was calculated by subtracting the amount of money lost on loss blocks from money won during gain blocks. In a single trial, there was no direct association between performance and the exact amount of money won or lost. Instead, the correctness of the response was indicated by abstract feedback (the respective money bag) together with the words “correct” or “incorrect!”. After each block, the outcome was calculated by the difference between correct responses during gain and incorrect responses during loss trials, for both c-dep and c-indep trials. Since performance on c-indep trials was close to ceiling, only ERs below 5% were rewarded the highest amount of 75 cents, with decreasing rewards as ERs increased. Overall, the achieved outcome was always greater than zero. At the end of the whole experiment, subjects again received feedback about the total amount of money won over the course of six blocks. Participants were instructed to respond as quickly and as accurately as possible.

Within a block of the AX-CPT, each trial started with a fixation cross (250 ms), followed by a motivational cue (1000 ms) indicating the incentive value (potential gain, potential loss, and neutral outcome) of the subsequent cue-probe combination. The motivational cue was followed by a blank interval (500 ms), the context cue (750 ms), and another blank (750 ms). The context cue indicated whether the following trial was c-dep or c-indep. C-dep and c-indep trials were randomly mixed within the task. Probes were presented for 5000 ms or until the subject responded, i.e., if the response was not given within 5000 ms, the trial was considered as a time-out. The probe was followed by another blank (500 ms). Finally, feedback (“correct,” “incorrect,” or “too slow”) was presented for 750 ms containing information about the response correctness and the achieved outcome. The inter-trial interval was 500 ms (see Schmitt et al., 2015).

### EEG Recording and Pre-processing

Participants were seated in a dimly lit, electrically shielded, and sound-attenuated chamber. EEG and electro-ocular activity (EOG) were recorded simultaneously by Brain Vision Recorder (Brain Products, Germany) with 59 Ag/AgCl active electrodes places in an elastic cap (extended international 10–20 system; Jasper, 1958). The left mastoid served as a reference and the ground electrode was placed at AFz. Impedances were kept below 20 kΩ. The EOG measured vertical eye movements from two electrodes above and below the right eye, and horizontal eye movements from the outer canthi of both eyes. EEG and EOG were low-pass filtered online (250 Hz), analog-to-digital converted (500 Hz SR), re-referenced to linked mastoids, and band-bass filtered offline from 0.01 to 30 Hz prior to statistical analysis. Whenever the standard deviation in a moving 200 ms time interval exceeded 30 μV in ocular electrodes or 20 μV in the representative electrode Cz, data were marked as artifacts. Recording epochs including eye-movements were corrected by using a linear regression approach (Gratton et al., 1983). The remaining artifacts were excluded after segmentation of the data by excluding the respective trials. Data pre-processing also included a visual screening for artifacts in all electrodes and trials with additional artifacts were removed before averaging. For visual presentation in **Figures 3** and **5**, the waveforms were low-pass filtered at 12 Hz. Offline EEG processing was done using EEProbe (ANT).

### Data Analysis

Practice blocks and trials with reaction times (RTs) faster than 100 ms were excluded from analysis (<0.1% of trials). The analysis of latencies was based on correct responses. The analysis of ERs included incorrect responses without time-outs. ERPs were recorded time-locked to the onset of the motivational cue, the context cue, and the probe and were analyzed at three midline electrodes over frontal (Fz), central (Cz), and parietal (Pz) areas. A 100 ms pre-stimulus baseline was used for all ERP averages. Note that we had to exclude one participant from the probe data due to less than 16 artifact-free trials for probe analysis. The selection of the time interval and the electrodes for statistical analyses of the EEG components was based on the literature and our previous ERP-analysis on the AX-CPT, together with visual inspection of peak latencies of the components obtained. In the motivational cue interval, we analyzed mean P2 amplitudes in a time window from 150 to 250 ms (cf. Olofsson et al., 2008), and mean P3 amplitudes in a time window from 500 to 700 ms after cue onset (cf. West and Alain, 2000b; Karayanidis et al., 2003; West et al., 2005; Friedman et al., 2008). In the context-cue interval, the analyses focused on the amplitude of the P3b and the CNV in time windows ranging from 450 to 650 ms and 1000 to 1500 ms after presentation of the context cue, respectively (cf. Karayanidis et al., 2003). Visual inspection of the ERPs in the probe epoch indicated that there seemed to be substantial component overlap between a centrally focused N450 and a parietally focused P3b. Because this makes it very difficult to draw conclusions regarding the underlying cognitive processes, we refrained from analyzing the probe-locked data.

In order to focus on motivational influences on context processing, and to analyze cue salience and valence effects, the effects of the motivational manipulation (for behavioral data and ERPs) were analyzed in terms of two *a priori* defined orthogonal contrasts: The first contrast (termed Salience effect) compared mean performance and ERPs on neutral blocks against the two motivational (gain and loss) blocks. The second contrast (termed Valence effect) compared mean performance and ERPs on loss vs. gain blocks. Consequently, for ERP data in the motivational cue interval, salience and valence effects were analyzed in ANOVAs including the additional factors Experimental Group (trial-wise vs. block-wise manipulation) and Anterior–Posterior (electrodes Fz, Cz, Pz). For behavioral data and ERPs in the context-cue interval, the effects of the Salience and Valence contrasts were analyzed in ANOVAs including the additional factors Experimental Group, Context Condition (c-dep, c-indep trials) and – for ERP data only – Anterior–Posterior (electrodes Fz, Cz, Pz). Additionally, to avoid unnecessary comparisons between electrode sites, the factor Anterior–Posterior was analyzed using repeated contrasts, i.e., Fz vs. Cz and Cz vs. Pz, that were defined *a priori*. For reasons of clarity, all significant (*p* < 0.05) and marginally significant (*p* < 0.10) effects are reported in the results section, non-significant effects are mostly omitted.

For all analyses, the alpha level was set to α = 0.05. Bonferroni–Holm corrections were applied on non-planned *post hoc* comparisons and the corrected *p*-values are reported. If necessary, Greenhouse–Geisser corrections for non-sphericity (Keselman and Rogan, 1980) were applied and epsilon corrected *p*-values are reported together with epsilon values (𝜀) and uncorrected degrees of freedom.

## Results

### Behavioral Data

**FIGURE 2:**
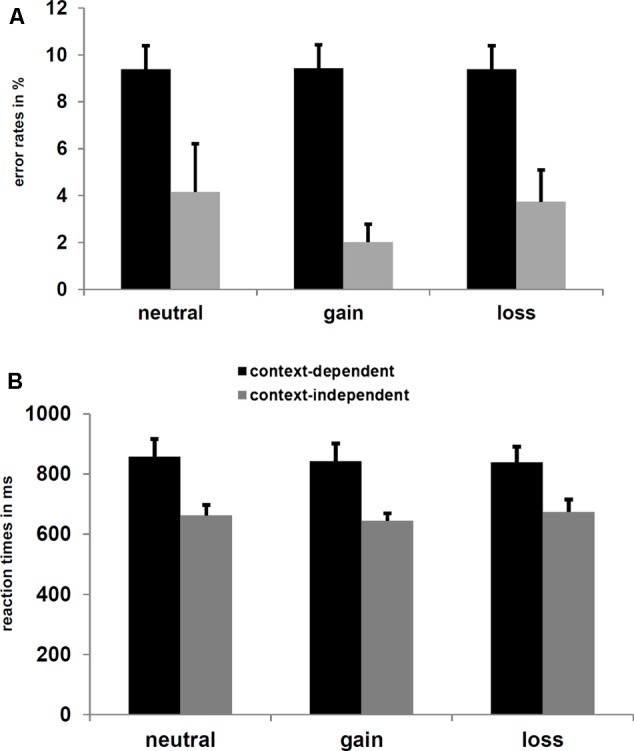
**(A)** Mean error rates (ER) and **(B)** reaction times for c-dep and c-indep trials on neutral, gain, and loss blocks. Error bars represent standard errors of the mean. Significant differences were found between c-dep and c-indep trials.

The ANOVAs with the factors Experimental Group (trial-wise vs. block-wise manipulation), Context (c-dep vs. c-indep), and the two planned contrasts reflecting Salience (motivational vs. neutral blocks) and Valence (gain vs. loss blocks) revealed a main effect of Context Condition for both, ERs, *F*(1,33) = 36.1, *p* < 0.001, $\eta_{p}^{2}$ = 0.52, and reactions times, *F*(1,33) = 57.1, *p* < 0.001, $\eta_{p}^{2}$ = 0.63, indicating higher ERs and longer latencies on c-dep than c-indep trials (see **Figure 2**). For ERs, there was also a marginally significant interaction of Experimental Group and Salience, *F*(1,33) = 3.0, *p* = 0.09, $\eta_{p}^{2}$ = 0.03. However, *post hoc* analyses did neither reveal a significant effect of Salience in one of the experimental groups (all *p*-values > 0.15), nor a significant effect of Experimental Group for salient or neutral cues (all *p*-values > 0.15).

### ERPs Locked to the Motivational Cue

#### P2

The ANOVAs with the factors Experimental Group, Anterior–Posterior, and the two planned contrasts reflecting Salience and Valence on the mean P2 amplitudes revealed significant effects of Experimental Group, *F*(1,33) = 5.6, *p* < 0.05, $\eta_{p}^{2}$ = 0.15, Anterior–Posterior (Fz/Cz contrast), *F*(1,33) = 8.5, *p* < 0.01, $\eta_{p}^{2}$ = 0.21, and of Salience, *F*(1,33) = 15.4, *p* < 0.001, $\eta_{p}^{2}$ = 0.32, and Valence, *F*(1,33) = 5.2, *p* < 0.05, $\eta_{p}^{2}$ = 0.14. It also resulted in interactions between Experimental Group and Salience, *F*(1,33) = 5.9, *p* < 0.05, $\eta_{p}^{2}$ = 0.15, Salience and Anterior–Posterior (Cz/Pz contrast), *F*(1,33) = 10.2, *p* < 0.01, $\eta_{p}^{2}$ = 0.24, Experimental Group and Valence, *F*(1,33) = 5.2, *p* < 0.05, $\eta_{p}^{2}$ = 0.14, and Experimental Group and Anterior–Posterior. To dissolve these interactions, separate analyses were calculated for the two experimental groups.

In the trial-wise manipulation group, we found a main effect of Salience, *F*(1,17) = 22.8, *p* < 0.001, $\eta_{p}^{2}$ = 0.57, denoting that salient trials elicited a larger P2 than neutral ones, and an interaction between Salience and Anterior–Posterior (Cz/Pz contrast), *F*(1,17) = 8.3, *p* < 0.05, $\eta_{p}^{2}$ = 0.33. This interaction was due to a larger central P2 for salient cues [P2 larger for salient than neutral cues at Cz: *F*(1,17) = 20.1, *p* < 0.001, $\eta_{p}^{2}$ = 0.54, P2 larger for salient than neutral cues at Pz: *F*(1,17) = 8.4, *p* < 0.05, $\eta_{p}^{2}$ = 0.33; P2 marginally larger at Cz than Pz for salient cues only, *F*(1,17) = 3, *p* = 0.09, $\eta_{p}^{2}$ = 0.17].

**FIGURE 3:**
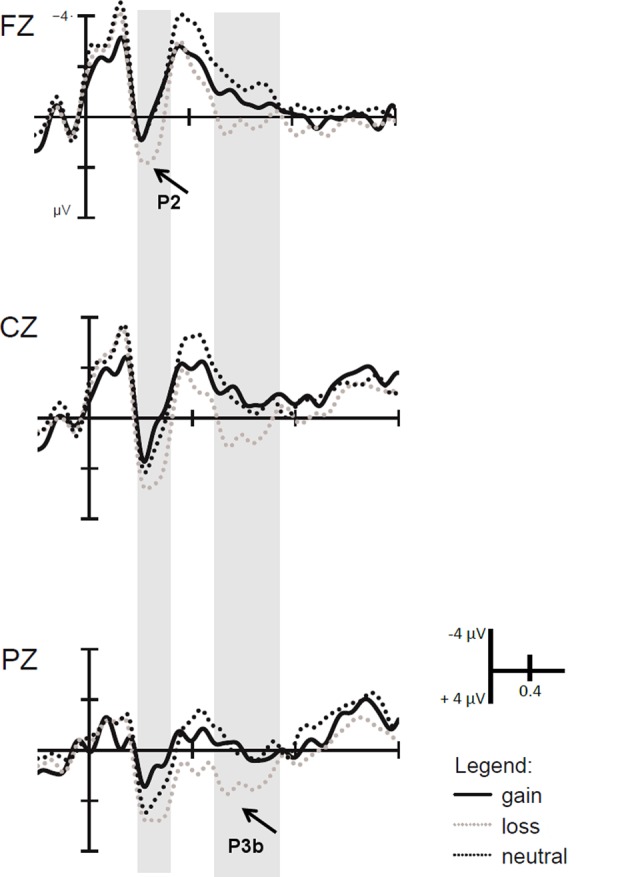
Event-related potentials (ERPs) time-locked to the motivational cue. Larger P2 amplitudes in the time window ranging from 150 to 250 ms were found for loss than gain cues. In the time window from 500 to 700 ms, larger P3b amplitudes were found for motivationally salient trials relative to neutral trials due to larger P3b amplitudes on loss trials.

**FIGURE 4:**
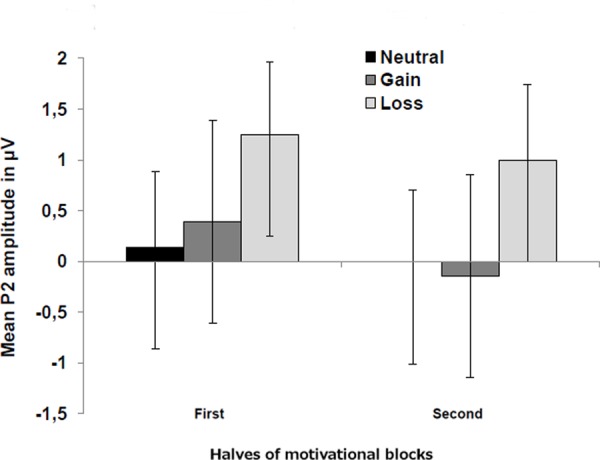
Mean P2 amplitudes for neutral, gain, and loss motivational cues presented separately for the first (including trials from 1 to 48) and second half (including trials from 49 to 96) of motivational blocks. Error bars represent standard errors of the mean. Results indicated that the P2 valence effect was stronger in the second half of blocks.

In the block-wise manipulation group, we found a main effect of Anterior–Posterior (Fz/Cz contrast), with larger P2 amplitudes at central than frontal sites, *F*(1,16) = 11.6, *p* < 0.01, $\eta_{p}^{2}$ = 0.42, as well as a significant effect of Valence, *F*(1,16) = 7.3, *p* < 0.05, $\eta_{p}^{2}$ = 0.31, indicating larger P2 amplitudes for loss vs. gain cues (see **Figure 3**). To check whether this P2 valence effect is reduced with repeated cue presentation, we additionally analyzed the first against the second half of trials within a block by means of *post hoc* tests. Results indicated that the P2 valence effect was marginally significant in the first half (*p* = 0.07), while a significant effect occurred in the second half of blocks, *F*(1,16) = 7.9, *p* < 0.025, $\eta_{p}^{2}$ = 0.33 (see **Figure 4**).

#### P3b

The ANOVAs with the factors Experimental Group, Anterior–Posterior, and the two planned contrasts reflecting Salience and Valence on the P3b amplitudes revealed a main effect of Anterior–Posterior, i.e., a parietal distribution of the P3b [larger P3b at Pz than Cz: *F*(1,33) = 6.6, *p* < 0.05, $\eta_{p}^{2}$ = 0.17; larger P3b at Cz than Fz: *F*(1,33) = 5.6, *p* < 0.05, $\eta_{p}^{2}$ = 0.14]. It also showed effects of Salience, *F*(1,33) = 17.0, *p* < 0.001, $\eta_{p}^{2}$ = 0.34, and Valence, *F*(1,33) = 18.5, *p* < 0.001, $\eta_{p}^{2}$ = 0.36, as well as an interaction between Experimental Group, Anterior–Posterior (Fz/Cz contrast), and Salience, *F*(1,33) = 4.6, *p* < 0.05, $\eta_{p}^{2}$ = 0.12, and a marginally significant interaction between Experimental Group, Anterior–Posterior (Fz/Cz contrast), and Valence, *F*(1,33) = 3.2, *p* = 0.08, $\eta_{p}^{2}$ = 0.09.

Separate analyses in the trial-wise and the block-wise Experimental Group showed significant effects of Salience, indicating a larger P3b for salient motivational than neutral cues [trial-wise: *F*(1,17) = 10.8, *p* < 0.01, $\eta_{p}^{2}$ = 0.39, block-wise: *F*(1,16) = 6.4, *p* < 0.05, $\eta_{p}^{2}$ = 0.29], and significant effects of Valence, i.e., a larger P3b amplitude for loss than gain cues [trial-wise: *F*(1,17) = 8.7, *p* < 0.01, $\eta_{p}^{2}$ = 0.34, block-wise *F*(1,16) = 10.5, *p* < 0.01, $\eta_{p}^{2}$ = 0.40]. To investigate why the interaction with Experimental Group occurred in the overall analysis, we additionally calculated *post hoc* analyses. These revealed that the salience effect in the block-wise group was due to a significant difference between loss and neutral cues, *F*(1,16) = 12.6, *p* < 0.025, $\eta_{p}^{2}$ = 0.44, but not between gain and neutral cues (*p* = 0.58, see **Figure 3**). In contrast, in the trial-wise group, there were significant differences between loss and neutral, *F*(1,17) = 14.7, *p* < 0.025, $\eta_{p}^{2}$ = 0.46, and gain and neutral cues, *F*(1,17) = 5.9, *p* < 0.025, $\eta_{p}^{2}$ = 0.44. This means that while there is a genuine effect of Salience and Valence in the trial-wise group, only loss cues induced a larger P2 and P3b during cue processing in the block-wise manipulation group.

### ERPs Locked to the Context Cue

#### P3b

**FIGURE 5:**
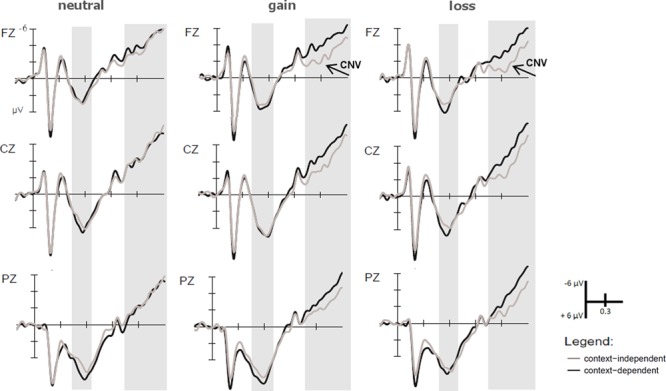
ERPs time-locked to the context cue. Marginally larger P3b amplitudes were found for gain than loss blocks in the time window ranging from 450 to 650 ms after context cue onset at central electrodes (Cz) only. In the time window from 1000 to 1500 ms, CNV amplitudes were larger for gain than loss cues at frontal (Fz) electrodes.

The ANOVAs with the factors Experimental Group, Context, Anterior–Posterior, and the two planned contrasts reflecting Salience and Valence on P3b amplitude showed significants main effects of Valence, *F*(1,33) = 5.9, *p* < 0.05, $\eta_{p}^{2}$ = 0.15, and Anterior–Posterior (Cz/Pz contrast), *F*(1,33) = 31.5, *p* < 0.001, $\eta_{p}^{2}$ = 0.49, and interactions between Anterior–Posterior (Fz/Cz contrast) and Experimental Group, *F*(1,16) = 12.6, *p* < 0.01, $\eta_{p}^{2}$ = 0.44, and Anterior–Posterior and Valence, *F*(1,33) = 8.3, *p* < 0.01, $\eta_{p}^{2}$ = 0.20. It also showed a marginally significant interaction between Anterior–Posterior (Fz/Cz contrast) and Salience, *F*(1,33) = 3.0, *p* = 0.09, $\eta_{p}^{2}$ = 0.08. Dissolving the interaction between Salience and Anterior–Posterior (Fz/Cz contrast) did not lead to any significant differences (all *p*-values > 0.34). Dissolving the interaction between Valence and Anterior–Posterior demonstrated that gain cues had larger P3b amplitudes than loss cues at central electrodes, *F*(1,34) = 8.4, *p* < 0.01, $\eta_{p}^{2}$ = 0.20 (see **Figure 5**).

#### Contingent Negative Variation (CNV)

The ANOVAs with the factors Experimental Group, Context, Anterior–Posterior, and the two planned contrasts reflecting Salience and Valence on CNV amplitudes showed a significant effect of Context, *F*(1,33) = 19.8, *p* < 0.001, $\eta_{p}^{2}$ = 0.38, indicating a larger (more negative) CNV for c-dep than c-indept trials, and of Anterior–Posterior [Fz > Cz contrast: *F*(1,33) = 17.1, *p* < 0.001, $\eta_{p}^{2}$ = 0.34; Cz > Pz contrast: *F*(1,33) = 48.8, *p* < 0.001, $\eta_{p}^{2}$ = 0.60], indicating a centrally focused CNV distribution. We also found a marginally significant effect of Experimental Group, *F*(1,33) = 3.8, *p* = 0.06, $\eta_{p}^{2}$ = 0.10 with larger CNV amplitudes for the trial-wise group. Additionally, significant interactions between Salience, Context, and Anterior–Posterior (Cz/Pz contrast), *F*(1,33) = 4.5, *p* < 0.05, $\eta_{p}^{2}$ = 0.12, and Valence, Experimental Group, and Anterior–Posterior (Fz/Cz contrast), *F*(1,33) = 4.7, *p* < 0.05, $\eta_{p}^{2}$ = 0.13, were obtained.

To dissolve the interaction including the factor Salience, separate ANOVAs were calculated for motivationally neutral and motivationally salient (gain and loss) trials. For salient trials, the CNV was larger at central than parietal electrodes for c-indep, *F*(1,34) = 34.4, *p* < 0.001, $\eta_{p}^{2}$ = 0.50, and c-dep trials, *F*(1,34) = 44.6, *p* < 0.001, $\eta_{p}^{2}$ = 0.58. Additionally, there was a context effect that was more pronounced at central as compared to parietal electrodes [Cz: *F*(1,34) = 13.6, *p* < 0.01, $\eta_{p}^{2}$ = 0.29, Pz: *F*(1,34) = 6.4, *p* < 0.05, $\eta_{p}^{2}$ = 0.16], as indexed by the effect sizes. In contrast, only a general effect of CNV distribution was found for neutral trials, with larger CNV amplitudes at central electrodes, *F*(1,34) = 45.5, *p* < 0.001, $\eta_{p}^{2}$ = 0.57.

To dissolve the interaction including the factor Valence, different ANOVAs were computed for each Experimental Group. These analyses revealed that for the block-wise group only, there was an interaction between Valence and Anterior–Posterior (Fz/Cz contrast), *F*(1,16) = 5.6, *p* < 0.05, $\eta_{p}^{2}$ = 0.26. *Post hoc* analysis showed that this interaction was due to a larger CNV for gain than loss cues at frontal electrodes, *F*(1,16) = 5.6, *p* < 0.05, $\eta_{p}^{2}$ = 0.26, as well as a larger CNV on central than frontal areas for loss cues, *F*(1,16) = 7.6, *p* < 0.05, $\eta_{p}^{2}$ine-formula> = 0.32 (see **Figure 5**).

### Summary of Results

In sum, our behavioral results showed a reliable context effect that was not modulated by gain and loss cues and did not differ between the experimental groups. In contrast, for the ERP data we did find differential Group effects. In the motivational cue epoch, salient gain and loss cues elicited a larger P2 and P3b than neutral cues and additionally loss cues elicited a larger P3b than gain cues in the trial-wise group. In contrast, in the block-wise group only the valence effects, i.e., a larger P2 and P3b for loss than for gain cues, remained significant. For the processing of the following context cue, however, we obtained a larger P3b to gain cues that was independent of Experimental Group. In the CNV after the context cue, we obtained a context effect, i.e., a larger CNV for c-dep than c-indep trials, for motivationally salient gain and loss trials in both experimental groups. Additionally, we obtained a larger CNV for gain than loss trials in the block-wise group only. Against our hypotheses, this effect did not interact with the context effect.

## Discussion

The aim of the present study was to examine whether a motivational mindset, i.e., a cognitive orientation toward positive (potential monetary gain) or negative (potential monetary loss) outcomes induced by a block-wise presentation of incentive cues, differentially influences the implementation of cognitive control in older adults. To this end, a block-wise presentation off incentive cues was compared with a trial-wise presentation. Cognitive control was measured by means of the AX-CPT in which older adults had to select their responses to a probe either dependent or independent of a context cue, thus creating a situation with changing demands on cognitive control. Moreover, we applied an ERP approach to examine (a) whether gain and loss cues are processed differently, and (b) whether these incentive cues affect the amount of cognitive control exerted during the following AX-CPT.

### Context Effects

The behavioral data showed longer latencies and higher ERs for c-dep than c-indep trials. This context effect reflects the fact that more cognitive control is necessary to successfully work on the more demanding c-dep trials (cf. Lenartowicz et al., 2010; D’Ardenne et al., 2012; Schmitt et al., 2014a). This behavioral context effect is in line with the ERP results, showing a larger CNV in the context cue epoch to c-dep than c-indep trials. The CNV context effect is thought to reflect the increased need of proactive task preparation in the more demanding c-dep trials and replicates our earlier findings in the AX-CPT without motivational incentives (Schmitt et al., 2014a).

Interestingly, motivational incentives (gains and losses) modulated the context effect in the CNV in the context-cue epoch irrespective of whether incentive cues were presented trial-wise or block-wise: In motivationally salient gain and loss trials, a context effect was obtained, while it was absent for neutral trials. This reflects that gain and loss cues increased task preparation in the more demanding c-dep trials. In the block-wise presentation group, we had expected that a positivity effect would emerge, i.e., that gains would enhance the context effect due to the induction of a motivational mindset. However, this was not found. One simple explanation for this lack of a positivity effect could be that older adults may have ignored the cues because they were constant across a block and thus, in principle, redundant. However, our ERP findings in the motivational cue and the context cue interval speak against this idea because they show that (a) the motivational incentive cues were processed differently than neutral cues (see Processing of Motivational Cues) and (b) they do influence cognitive processes during task preparation even after repeated presentation (see Motivational Influences on Cognitive Processing during the AX-CPT). This implies that trial-wise presentation of incentive cues affects cognitive processing in a different way than block-wise presentation of incentive cues, which evokes a more transient motivational mindset. Thus, a more likely explanation is that block-wise in comparison to trial-wise presentation reduces working memory load for older adults to a level that allows them to perform the task similar to when no incentive cues are presented. This is, however, not reflected in a modulation of context effects.

### Processing of Motivational Cues

In the motivational cue epoch, we found that P2 amplitudes were larger after salient gain and loss cues than after neutral cues in the trial-wise group, while they were larger for loss than gain cues in the block-wise group. P3b amplitudes were larger for motivationally salient than neutral cues in the trial-wise group and also larger after loss than gain cues. In the block-wise group, in contrast, we did not obtain a salience effect, but a larger P3b for loss than gain cues. The P2 is known to be associated with a rapid allocation of attention toward a stimulus (Luck and Hillyard, 1994). It has not only been observed in purely cognitive paradigms, but also in terms of an increased orienting of attention toward emotional stimuli (e.g., Ito et al., 1998; Carretié et al., 2001; Huang and Luo, 2006; Kanske and Kotz, 2007; Kanske et al., 2011). The P3b has been linked to the strategic updating of an internal model of the environment by task-relevant information and the relevance ascribed to the motivational cue (Donchin and Coles, 1988; Briggs and Martin, 2009; Krebs et al., 2013). Thus, when presented with incentive cues in a block-wise fashion, older adults seem to allocate more attention to loss cues and invest more in updating task-relevant information after having been presented with a loss cue.

In line with this result, there is evidence that the cognitive processes reflected in the P2 and P3b can be applied relatively flexible. For example, Kanske and Kotz (2007) observed a larger P2 amplitude for emotionally positive as opposed to neutral words while younger adults performed a lexical decision task (see also Kanske et al., 2011). This attention capture effect is not uniformly found toward positive but also toward negative stimuli (e.g., the presentation of negative IAPS pictures; Ito et al., 1998; Carretié et al., 2001; Huang and Luo, 2006). In a similar vein, Zinchenko et al. (2015, 2017) demonstrated that positive as well as negative emotional stimuli lead to increased attention allocation as reflected in an enhanced P200 during conflict processing. Moreover, it has been found that in conditions of heightened state anxiety, there is increased attention allocation to stimuli in a threatening context (Mercado et al., 2006). Although these studies examine emotional rather than motivational manipulations, they show that the attention allocation as reflected in the P2 can be applied relatively flexible, depending on task characteristics. Similarly, it has been demonstrated that P3b amplitude can be influenced by subjective processes of stimulus categorization (Mecklinger and Ullsperger, 1993). Together, this evidence suggests that orienting of attentional resources and updating of task-relevant information can be flexible and depend on task and or person characteristics.

In the present study, inducing a motivational mindset via block-wise presentation of motivational incentives increased the rapid allocation of attentional resources (as reflected in the larger P2 for losses than gains) and the updating of task-relevant information (as reflected in the larger P3b for losses than gains) toward cues signaling potential losses. In the experimental group with trial-wise presentation of incentive cues, this valence effect was only found for the P3b. However, in this group, a salience effect was found in the P2 and the P3b, i.e., older adults paid more attention to all motivationally salient gain and loss cues. As outlined above, this operationalization probably imposed higher demands on working memory resources than a block-wise manipulation, especially for older adults. Thus, it could be speculated that working memory demands might be an additional task characteristic that changes the rapid allocation of attentional resources toward motivational cues. In the case of a high working memory load, older adults might mainly classify the different cues into “salient” vs. “neutral,” while under conditions in which they have more resources left, losses might play a more important role. However, this idea needs to be tested more directly in future research by explicitly manipulation working memory load.

Because it has been demonstrated that this rapid allocation of attentional resources decreases with repetition of the same stimulus (Luck and Hillyard, 1994), we additionally investigated in a control analysis whether this valence effect diminishes over the course of time when participants become familiar with the stimuli. This analysis found quite the reverse pattern, namely that the effect was stronger in the second half of the motivational block. Thus, unlike for the repeated presentation of motivationally neutral stimuli, the rapid allocation of attentional resources toward cues indicating potential losses did not abate over time in older adults. This is especially interesting because, in principle, the motivational cues were redundant and hence could mean that they were not able to ignore the negative value of the loss cues specifically.

### Motivational Influences on Cognitive Processing during the AX-CPT

Although motivational cues did not modulate context effects (except for the salience effect in the CNV, see section “4.1 Context Effects” and below), they did influence cognitive processing in the AX-CPT. However, in contrast to the motivational cue epoch where losses were of greater importance, ERP data in the context cue epoch of the AX-CPT showed stronger reactivity to motivational gain cues. Here, we found a larger P3b in the context cue epoch after gains than losses for both experimental groups. Note, however, that when the trial-wise group of older adults was compared to a trial-wise group of younger adults in our previous study (Schmitt et al., 2015), a larger P3b had been found for salient than neutral trials. This salience effect did not reach significance in the present comparison of the two older participant groups.

In the CNV of the context cue epoch, we found larger amplitudes for potential gains and losses in both experimental groups. This salience effect interacted with context (larger CNV for c-dep than c-indep trials after salient cues, see section “Context Effects” above). Only in the block-wise experimental group an additional valence effect emerged, i.e., in this group gain trials elicited a larger CNV than loss trials. These results indicate that (a) during actual task preparation (as opposed to motivational cue processing), potential gains seem more important than potential losses and (b) in the block-wise group, this effect is even stronger than in the trial-wise group because they not only show more updating of task-relevant information (as reflected in the P3b) but also more maintenance and response preparation (as reflected in the CNV) under conditions with potential gains. Unfortunately, due to component overlap, we were not able to analyze the influence of motivational incentives on response-related processes in the probe interval.

Together, our results from the context cue epoch demonstrate that incentives can lead to the implementation of enhanced task preparation in older adults. This adds to previous research showing that older adults control style can be changed to become more proactive by extended practice on the task (Braver et al., 2009). Our results also show that trial-wise and block-wise presentation of incentive cues change preparatory processing in different ways: while during block-wise presentation only gain cues influence proactive processing, the evidence from the trial-wise presentation group is mixed with gains enhancing working memory updating (P3b) but gains and losses enhancing maintenance and response preparation (CNV). As described in detail above, a trial-wise presentation of the motivational cues imposes high demands on working memory because the cues are not redundant. This may have resulted in a different categorization of the cues and thus a different focus.

To sum up, the results from the motivational cue epoch indicate that older adults are more sensitive to the presentation of a loss cue. This would speak in favor of the notion that the prevention of losses is more relevant to older adults than the receipt of gains as would be predicted by the model of Selection, Optimization, and Compensation (SOC) by Baltes and Baltes (1990; for a similar view, see Brandtstädter, 2009). In contrast, the pattern of results from the context cue epoch imply that older adults invest more cognitive resources under conditions in which potential gains can be obtained. This result would be expected according to the age-related positivity effect as assumed by the SST (e.g., Mather and Carstensen, 2005). This pattern of results seems contradicting at first glance, however, it indicates several important issues that need further consideration. First, the SST and the SOC model were developed in a different research context that aimed at explaining a means to regulate emotional well-being and goal motivation in old age and might not be easily transferred to cognitive control processes. Second, it hints at the possibility that the processing of motivational cues and the allocation of attentional and control processes is more flexible than would be expected by a age-related positivity effect or loss-prevention mindset that are constantly in effect, namely that it is dependent on the task, the person, and the availability of cognitive resources. This idea is consistent with recent behavioral studies showing that older adults prioritize the processing of negative over positive information when it holds survival value (such as the processing of negative emotional faces, Reed and Carstensen, 2012) and that a positivity effect is not obtained in older adults when avoiding negative information has detrimental effects while attending to negative information is behaviorally adaptive (Reed and Carstensen, 2012). Third, it hints at a possible dissociation between the immediate (and probably more automatic) reaction to stimuli holding motivational value vs. the more strategic and goal-directed use of controlled processing. In this sense, the present study found a strong initial reaction to stimuli signaling potential losses that may serve a kind of arousal function and support orienting of attention to an alerting stimulus. Although they are, in principal, redundant, older adults are not able to ignore these potential loss stimuli even after prolonged presentation. Hence, the processing of the loss cues may indicate a genuine processing bias in older adults. On the other hand, older adults show increased investment in preparatory processes when gains can be obtained by optimal performance and sufficient cognitive resources are available to perform the task at hand.

### Limitations and Future Research Directions

The present findings demonstrate that older adults react more strongly to cues signaling potential losses, but invest more cognitive resources during the preparation for an upcoming probe stimulus in conditions where gains can be obtained when a motivational mindset is induced by block-wise presentation of gain, loss or neutral cues. However, it should be noted that the present study did not involve a younger control group, although by definition, the age-related positivity effect and the loss aversion of older adults usually is examined as a relative difference between older and younger people (Baltes and Baltes, 1990; Reed and Carstensen, 2012). Therefore, we can only make conclusions about the impact of a motivational mindset in older adults. Whether younger and older adults differ in valence processing during block-wise presentation of motivational cues needs to be investigated in future work.

Recent research has begun to investigate the time-course of processing incentive and task-cue information on cognitive control to investigate its additive or interactive nature (Chiew and Braver, 2015). Regarding the present study, we found no actual interaction between incentive and task-cue information in the block-wise group. However, there is evidence from younger adults that during trial-based motivational cueing, the timing of informative and incentive cue presentation critically affects its impact on proactive control performance (Chiew and Braver, 2015). Hence, future work may more thoroughly manipulate the timing and nature of combined reward and context cues in an ERP approach and extend this relationship toward the understanding of cognitive aging. Besides, both incentive processing and the updating of contextual information seem to rely on dopaminergic activation. As we only used behavioral and ERP data to investigate salience and valence effects, it might therefore be necessary to use molecular imaging techniques to link the processing of motivational cues to salience and valence effects on the neuronal level. At this point, is important to note that most aging studies so far only applied reward motivations, but not penalties on cognitive control tasks, so that the precise nature of age differences in salience and valence effects remains unknown (cf. Chiew and Braver, 2011; Ferdinand and Kray, 2013).

## Conclusion

Taken together, the findings of the present study show that when motivational cues signal that potential gains, losses, or neutral outcomes can be obtained depending on performance in the following task, older adults initially react more strongly to cues signaling potential losses, but then invest more cognitive resources during the preparation for an upcoming probe stimulus in conditions with potential gains. Additionally, presenting incentive cues on a trial-wise or block-wise basis makes a difference. During block-wise presentation valence effects are consistently found. The effects during a trial-wise presentation mode are rather mixed and salience as well as valence effects can be obtained. This is probably due to the trial-wise presentation being more demanding. By this, the study furthers our understanding of how motivational incentives modulate task-related processing and the implementation of cognitive control. It also emphasizes the need for further studies focusing on task characteristics and individual differences that influence the availability of cognitive resources in cognitive-affective interactions.

## Ethics Statement

This study was carried out in accordance with the recommendations of the Declaration of Helsinki and approved by the local ethics committee of Saarland University. All subjects gave written informed consent and were paid 8€ per hour with an additional reimbursement based on their money won during the experiment.

## Author Contributions

HS, NF, and JK conceived the research design of the study. HS and NF carried out the statistical analyses and interpretations. All authors contributed to the writing of the manuscript.

## Conflict of Interest Statement

The authors declare that the research was conducted in the absence of any commercial or financial relationships that could be construed as a potential conflict of interest.
